# Superior outcome of upfront allogeneic hematopoietic cell transplantation versus hypomethylating agent induction in myelodysplastic syndrome

**DOI:** 10.1038/s41409-024-02365-5

**Published:** 2024-07-09

**Authors:** Jan Christian Schroeder, Lucas Mix, Philipp Faustmann, Jan Frederic Weller, Adrian Fehn, Laurent Phely, Andreas Riedel, Wichard Vogel, Christoph Faul, Claudia Lengerke, Wolfgang Andreas Bethge

**Affiliations:** Department for Internal Medicine 2, Hematology, Oncology, Clinical Immunology and Rheumatology, University Hospital Tuebingen, Tuebingen, Germany

**Keywords:** Myelodysplastic syndrome, Stem-cell therapies, Disease-free survival

Myelodysplastic syndromes (MDS) are heterogeneous diseases with variable clinical outcome and an inherent risk of progression to acute myeloid leukemia (AML). Allogeneic hematopoietic cell transplantation (alloHCT) is the standard curative treatment [1] but the requirement for pre-transplant induction therapy remains unclear in MDS. In 2022, the new entity MDS/AML was introduced by the European LeukemiaNet (ELN) 2022 guidelines to highlight the overlapping biology irrespective of precise blast counts [2] in high-risk MDS and incipient AML and underscore the significance of genetic aberrations over morphological findings.

However, in the current standard of care, bone marrow (BM) blast counts still strongly influence therapy choice. While patients with low blast counts may receive upfront alloHCT, patients with blast counts >10% [1] or MDS/AML are rather pre-treated with remission-inducing agents before alloHCT. In elderly or frail patients, hypomethylating agents (HMA) are often preferred over intensive chemotherapy due to lower toxicity [3]. However, MDS-IB2 or MDS/AML can also be treated with upfront alloHCT [4]. Retrospective data support this concept [5], but prospective evidence from randomized trials is missing.

Here we present the outcome of 109 MDS patients undergoing alloHCT at our center between 2010 and 2022 with or without HMA pre-treatment (Figure S1). The median age was 59 years and 49 of 109 patients (45.0%) were female. 40 of 109 (36.7%) had MDS-LB (<5% BM blasts), 26.6% MDS-IB1 (5–9% blasts), and 36.7% MDS-IB2 (10–19% blasts). 50.4% had intermediate-, poor-, or very poor-risk cytogenetic aberrations according to the IPSS-R cytogenetic risk category, and allocation to alloHCT was in 95.4% based on diagnosis of high-risk disease and on intermediate IPSS and/or refractory cytopenia in the remainder.

65.1% (71/109) of the patients received upfront alloHCT (upfront group) and 34.9% HMA-based treatment (HMA group). 92.1% (33/38) HMA group patients received more than one cycle azacytidine, with a median of 4 cycles, and 6/109 received HMA in a prospective trial. In the remaining patients, upfront alloHCT was preferred if donor was rapidly available and no disease acceleration was observed until alloHCT was scheduled. In the upfront group, 68 of 71 patients (95.8%) received upfront alloHCT without prior disease-specific treatment (two lenalidomide and one luspatercept).

HMA group patients were older (median 63 vs. 56 years), had lower Karnofsky index and higher BM blast counts at diagnosis when compared to upfront group patients (Table S1). No differences were observed with respect to IPSS-R or cytogenetics. At alloHCT, 13 of 33 HMA group patients (39.4%) achieved a complete response (CR), resulting in comparable blast counts with the upfront group (Fig. S2), and time from diagnosis to alloHCT was comparable (*p* = 0.593). AlloHCT was performed with reduced intensity conditioning in 99 of 109 (90.8%) cases, 27 (27.3%) of which with sequential conditioning. In 23 (21.1%) cases, a mismatched unrelated or haploidentical donor was used.

After a median follow-up of 63 months, Kaplan–Meier estimated probabilities for 5-year overall survival (OS), relapse-free survival (RFS), non-relapse mortality (NRM), and relapse incidence were 55% [95% CI 45.4–66.8%], 45.5% [95% CI 36.3–57.2%], 17% [95% CI 10–25%], and 39% [95% CI 29–49%], respectively (Fig. S3). Unexpectedly, both 5-year OS (37.4% vs. 65.8%, *p* = 0.023) and RFS (30.9% vs. 53.4%, *p* = 0.022) were shorter in the HMA versus the upfront group (Figs. 1a and S4). The HMA group showed higher 5-year relapse incidence (53% vs. 31%, *p* = 0.03) but no difference in 5-year NRM (16% vs. 18%, *p* > 0.9) versus the upfront group (Figs. 1b and S4). Intriguingly, 27 (24.8%) patients who received HMA-based salvage therapy for relapse showed by trend shorter OS and lower CR/CRi rates if initially treated with HMA prior to alloHCT (2-year OS 17.9% vs. 51.7%; *p* = 0.064; CR/CRi rate 21.4% vs. 53.8%, *p* = 0.12; Fig. 1c). This suggests poorer disease control as the main mechanism impairing long-term outcome in patients receiving HMA prior to alloHCT in line with previous data [6].

**Fig. 1:**
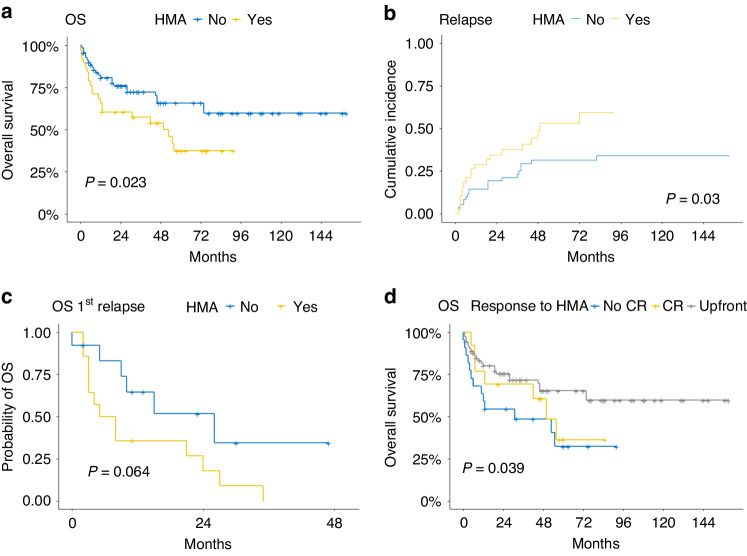
Inferior alloHCT outcomes after remission induction with HMA. Comparison of time-dependent outcome variables according to remission induction with hypomethylating agents (HMA) using Kaplan-Meier estimator and log-rank test. Relapse incidence and non-relapse mortality were analyzed as competing risks and tested for significant differences between groups by Gray’s test. **a** 5-year overall survival (OS) for the HMA vs. the upfront group: 37.4% vs. 65.8% (*p* = 0.023), *n* = 109. **b** 5-year relapse incidence for the HMA vs. the upfront group: 53% vs. 31% (*p* = 0.03), *n* = 109. **c** 2-year OS from first relapse post allogeneic hematopoietic cell transplantation (alloHCT) for the HMA vs. the upfront group: 17.9% vs. 51.7%; *p* = 0.064. Only patients receiving salvage treatment including HMA were analyzed, *n* = 27. **d** OS according to marrow complete response (CR) to HMA treatment or upfront HCT, *n* = 75 according to availability of BM response assessment before alloHCT.

Multivariate regression analysis revealed cytogenetic risk and pre-treatment with HMA as predictors of inferior OS and RFS and higher relapse incidence (Fig. S5). As expected, the strongest effects were observed for cytogenetic risk (HR 6.06 [95% CI 2.49–14.74, *p* < 0.001] for OS, 4.12 [95% CI 1.93–8.79, *p* < 0.001] for RFS, 6.69 [95% CI 2.87–15.61, *p* < 0.001] for relapse incidence), but HMA induction remained associated with inferior OS (2.06 [95% CI 1.08–3.95, *p* = 0.029]) and RFS (1.95 [95% CI 1.09–3.49, *p* = 0.025]), and higher relapse incidence (2.09 [95% CI 1.05–4.15, *p* = 0.035]) in the multivariate regression analysis. In addition, propensity score-matching confirmed inferior OS and RFS in the HMA group (Fig. S6).

In contrast to cytogenetic risk and pre-treatment strategy, blast counts did not impact OS (HR 1.62 [95% CI 0.69–3.82, *p* = 0.273]), RFS (HR 1.29 [95% CI 0.60–2.75, *p* = 0.511]) or relapse incidence (HR 1.77 [95% CI 0.73–4.29, *p* = 0.2]) (see Fig. S5). Furthermore, outcomes of 13 of 33 (34.2%) patients achieving CR after HMA were not superior to patients receiving upfront alloHCT (Fig. 1d).

Remission at alloHCT is a known predictor of favorable outcomes in MDS and AML, based on retrospective data of patients who received remission induction in most cases [7]. In contrast, similar to our results, retrospective analyses of MDS and secondary AML patients do not indicate improved outcomes in patients achieving CR when compared to upfront alloHCT [6, 8]. Furthermore, CR rates after HMA treatment of MDS patients in clinical trials were typically less than 20% [9]. At the same time, a recent prospective trial described a drop-out rate under HMA treatment of over 30% [10], and a retrospective analysis of 157 MDS patients reported drop-out rates prior to alloHCT of 5.6% for upfront alloHCT versus 29% for ICT or HMA induction, respectively [11].

Another common consideration is the application of HMA as bridge-to-transplant in MDS-IB. Interestingly, despite a median time to HCT of 7 months in our cohort, the rates of MDS progression were similar in both treatment groups and transformation to AML before alloHCT occurred in only 5 of 109 (4.6%) patients (Fig. S7). This is supported by results from a recent phase III trial that did not find superior response rates and 4-year OS in relapsed or refractory AML patients receiving salvage chemotherapy for remission induction prior to alloHCT versus upfront alloHCT [12].

Together, these findings reinforce that upfront alloHCT is a feasible and perhaps superior strategy for the treatment of MDS-IB or MDS/AML. This is of interest since current clinical trials intensively compare different induction regimens (e.g., the VERONA trial comparing HMA only with HMA/Venetoclax, the PALOMA trial comparing induction with 7 + 3, HMA or CPX-351) but no prospective study involves further comparison with upfront alloHCT. Clearly, at the proliferative end of the MDS/AML spectrum, remission induction will be necessary to prevent overwhelming disease progression. However, the use of BM blast count alone to make this distinction seems inadequate. Instead, we propose that genetic features and biomarkers beyond mere blast counts need to be considered. Due to the inherent limitations of retrospective studies, we are currently preparing a prospective randomized clinical trial to compare upfront alloHCT with alloHCT after remission induction in MDS, MDS/AML, and oligoblastic AML.

### Supplementary information

Supplemental material

## Data Availability

Data are available on basis of institutional review board-approved data request in alignment with applicable data protection regulations.
