# Supplementary material for: Superior outcome of upfront allogeneic hematopoietic cell transplantation versus hypomethylating agent induction in myelodysplastic syndrome
Source: Bone Marrow Transplant. 2024 Jul 9;59(9):1332–4. doi: 10.1038/s41409-024-02365-5 (PMC11368806; doi:10.1038/s41409-024-02365-5)
Supplement: Supplementary file 1 — Supplemental material [file 41409_2024_2365_MOESM1_ESM.pdf]

Superior outcome of upfront allogeneic hematopoietic cell transplantation versus hypomethylating agent induction in myelodysplastic syndrome

Jan Christian Schroeder[1], Lucas Mix[1], Philipp Faustmann[1], Jan Frederic Weller[1], Adrian Emanuel Fehn[1], Laurent Phely[1], Andreas Riedel[1], Wichard Vogel[1], Christoph Faul[1], Claudia Lengerke[1]*, Wolfgang Andreas Bethge[1]*

[1] Department for Internal Medicine 2, Hematology, Oncology, Clinical Immunology and Rheumatology, University Hospital Tuebingen, Tuebingen, Germany

* These authors contributed equally to this work

Supplementary Material

Supplementary methods

Patients

The analysis includes 109 patients consecutively transplanted for MDS between 2010 and 2022 at our center. Six of 109 received HMA prior to alloHCT in a prospective trial[1] (NCT01404741). In the remaining patients, decision to induce remission with HMA prior to alloHCT versus upfront alloHCT was made based on treating physician´s assessment and an interdisciplinary treatment board. Upfront alloHCT was preferred if a) donor was rapidly available and b) no disease acceleration was observed until alloHCT was scheduled. We retrospectively analyzed patient characteristics and outcome with data lock in July 2023. The median follow-up time was 63 months, and the completeness of follow-up was 82.3%. The investigation was conducted according to the declaration of Helsinki, European data protection regulations and approved by our institutional review board.

Definitions

Diagnoses were based on the 2022 edition of the WHO classification of myeloid neoplasms[2], with the exception that MDS were categorized within the group "MDS, morphologically defined" due to a lack of data on *SF3B1* and *TP53* mutations in 67 of 109 patients (61.5%). Risk classification was based on the Revised International Prognostic Scoring System (IPSS-R) for Myelodysplastic Syndromes[3], which was also used for cytogenetic risk stratification. Complete response (CR) in the BM was defined according to the most recent international working group response criteria for MDS[4] and CR with incomplete hematologic recovery (CRi) followed ELN 2022 recommendations[5]. HLA-matching of stem cell donors was based on a minimum resolution of 8 alleles, with matched donors representing a match of 8/8 or better.

Statistical analysis

Descriptive statistics used frequencies and percentages for categorical variables and median, mean, range and standard deviation for continuous variables. Differences between groups were assessed by Fisher's exact test. Probabilities of OS and RFS were analyzed using Kaplan-Meier estimator and log-rank test. Relapse incidence and non-relapse mortality (NRM) were estimated with relapse and death as competing events using Gray's test. OS was defined as the time from alloHCT to death, and RFS as time from alloHCT to relapse or death, depending on which event occurred first. NRM was defined as death after alloHCT without prior relapse. Uni- and multivariate (MV) Cox proportional hazards regression was used to analyze predictive factors for OS and RFS. Uni- and multivariable (MV) cause-specific hazards regression was used to analyze predictive factors for NRM and relapse. Dummy coding was used for categorical variables, while age and BM blasts were analyzed as continuous variables. Variables for the MV models were selected based on plausibility, significance of differences in univariate analysis, and maximization of Akaike's information criterion. Results are presented as hazard ratio (HR) with 95% confidence interval (CI), all tests and CIs being two-sided. 1:1 nearest neighbor propensity score matching without replacement was performed based on significant intergroup differences and parameters underlying treatment group assignment. The level of significance was set at 0.05 for all tests. Statistical analysis and visualization of survival and incidence curves were performed using R 4.3.0[6] and RStudio 2023.03.0[7].

Supplementary tables

Table S1: Demographic, clinical and treatment parameters of the HMA and upfront alloHCT groups.

Fisher's exact test was used to test for differences in the composition of substrata between treatment groups, n=109. BM blasts, bone marrow blast percentage at diagnosis. Conditioning regimens: BuCy, Busulfan/Cyclophosphamide; FLAMSA, fludarabine/amsacrine/cytarabine intensified sequential conditioning, followed by fludarabine/busulfan; FluBu, Fludarabine/Busulfan; FluTreo, Fludarabine/Treosulfan. HCT-CI, Hematopoietic cell transplantation-specific comorbidity index. HMA, hypomethylating agent. IPSS-R, Revised International Prognostic Scoring System. SD, standard deviation.

Parameter	Strata	All	Upfront	HMA	p value
Total, n (% of all patients)		109 (100.0)	71 (65.1)	38 (34.9)	
Age in years, mean (SD)		57.7 (10.4)	55.1 (10.6)	62.4 (8.4)	**<0.001**
Age in years, median (range)		59 (26-77)	56 (26-77)	63 (36-76)	
Sex, n (%)	Male	60 (55.0)	41 (57.7)	19 (50.0)	0.545
	Female	49 (45.0)	30 (42.3)	19 (50.0)	
BM blasts, n (%)	< 5%	40 (36.7)	34 (47.9)	6 (15.8)	**0.001**
	5 - 9%	29 (26.6)	18 (25.4)	11 (28.9)	
	10 - 19%	40 (36.7)	19 (26.8)	21 (55.3)	
IPSS-R, n (%)	Very low	1 (1.0)	1 (1.4)	0 (0.0)	0.612
	Low	8 (7.6)	7 (10.1)	1 (2.8)	
	Intermediate	26 (24.8)	18 (26.1)	8 (22.2)	
	High	29 (27.6)	17 (24.6)	12 (33.3)	
	Very high	41 (39.0)	26 (37.7)	15 (41.7)	
Cytogenetic risk, n (%)	Good	54 (49.5)	35 (49.3)	19 (50.0)	0.961
	Intermediate	24 (22.0)	16 (22.5)	8 (21.1)	
	Poor	15 (13.8)	9 (12.7)	6 (15.8)	
	Very poor	16 (14.7)	11 (15.5)	5 (13.2)	
Conditioning, n (%)	FluTreo	39 (35.8)	30 (42.3)	9 (23.7)	0.110
	FluBu	28 (25.7)	15 (21.1)	13 (34.2)	
	BuCy	10 (9.2)	8 (11.3)	2 (5.3)	
	FLAMSA	27 (24.8)	14 (19.7)	13 (34.2)	
	Other	5 (4.6)	4 (5.6)	1 (2.6)	
Donor, n (%)	Matched	86 (78.9)	57 (80.3)	29 (76.3)	0.631
	Mismatched	23 (21.1)	14 (19.7)	9 (23.7)	
Karnofsky index, n (%)	100	38 (34.9)	32 (45.1)	6 (15.8)	**0.003**
	≤ 90	71 (65.1)	39 (54.9)	32 (84.2)	
HCT CI, n (%)	0	60 (55.0)	40 (56.3)	20 (52.6)	0.840
	≥ 1	49 (45.0)	31 (43.7)	18 (47.4)	
Months to HCT, median (range)		7 (2-93)	6 (2-93)	8 (2-81)	0.593

Supplementary figures

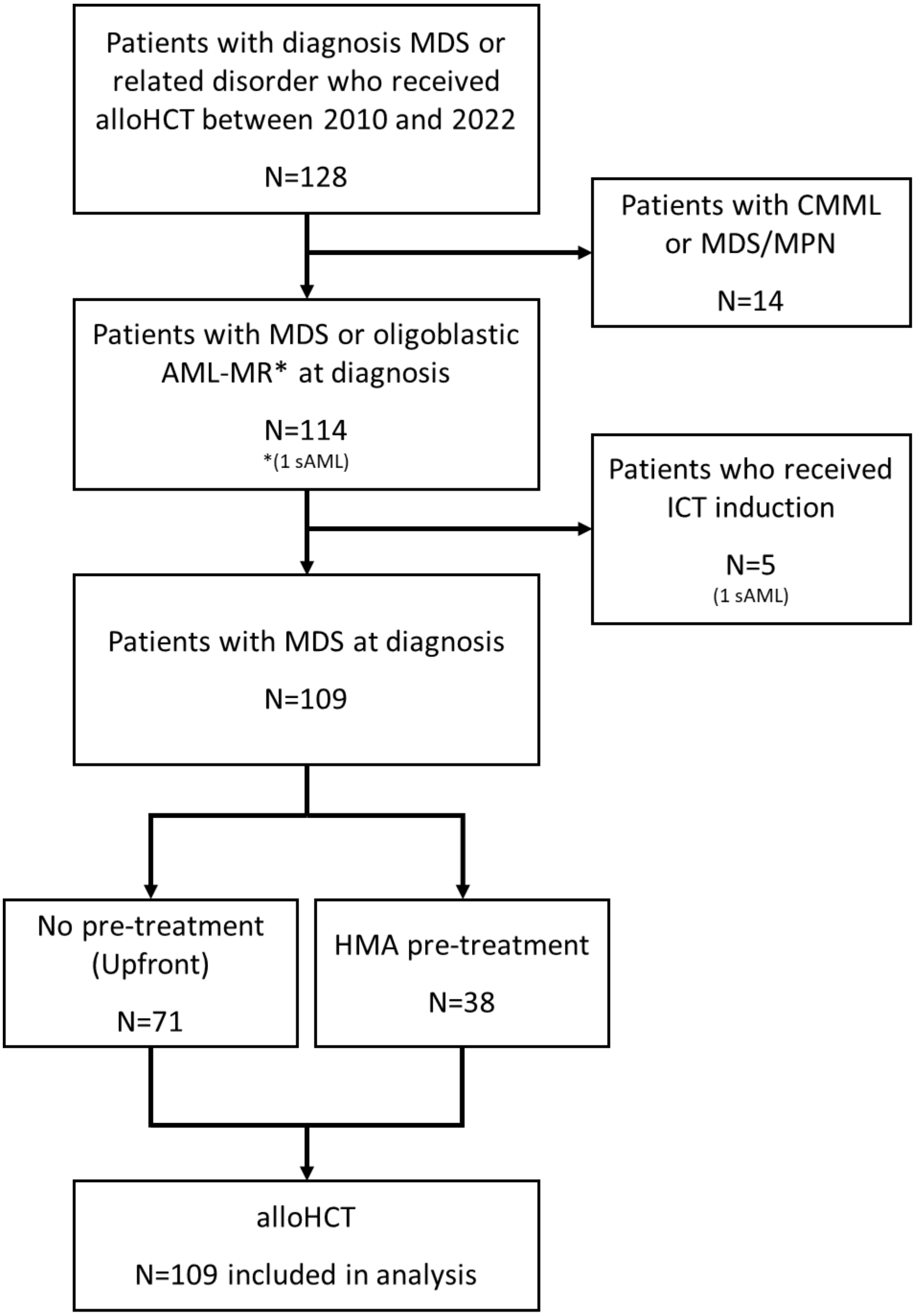

Figure S1: Study cohort

All MDS patients who received allogeneic hematopoietic cell transplantation (alloHCT) between 2010 and 2022 at our center were identified. 14 patients with diagnoses chronic myelomonocytic leukemia (CMML) or mylodysplastic/myeloproliferative neoplasm (MDS/MPN) and 5 patients who had received intensive chemotherapy (ICT) were excluded. 109 patients remained, 38 of whom had received pre-treatment with hypomethylating agents (HMA) prior alloHCT, and 71 of whom had received upfront alloHCT. One patient who had received ICT had acute myeloid leukemia, myelodysplasia-related (AML-MR) at diagnosis according to our standardized evaluation, but this patient was not included in the final analysis.

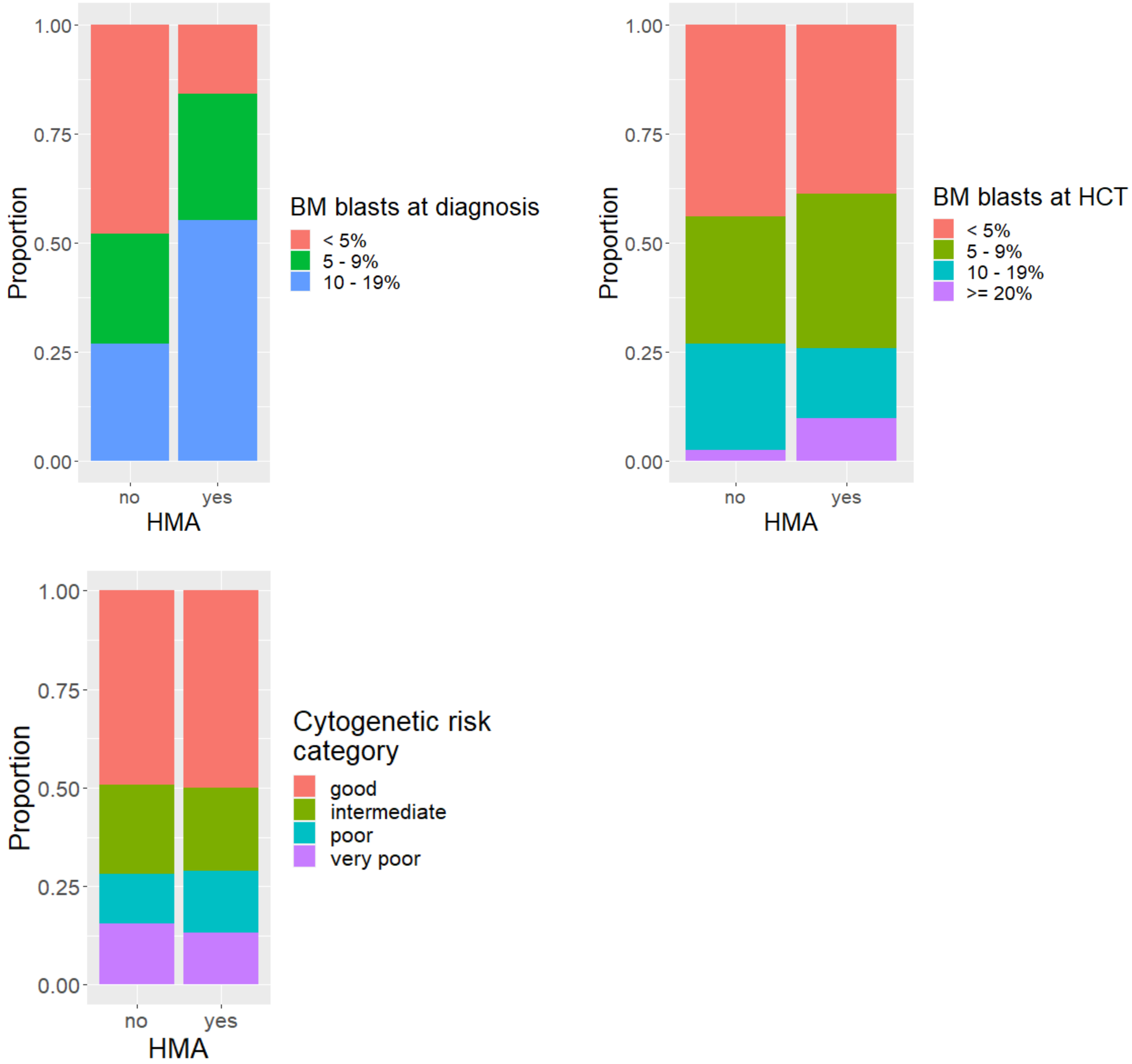

Figure S2: Comparison of BM blast counts and cytogenetic risk between groups

Stratification of patients according to remission induction with hypomethylating agents (HMA). Bone marrow (BM) blast count at diagnosis (left panel), n=109, BM blast count at HCT (right panel), n=72 patients with available BM blast count pre-alloHCT. Cytogenetic risk category (bottom panel), n=109.

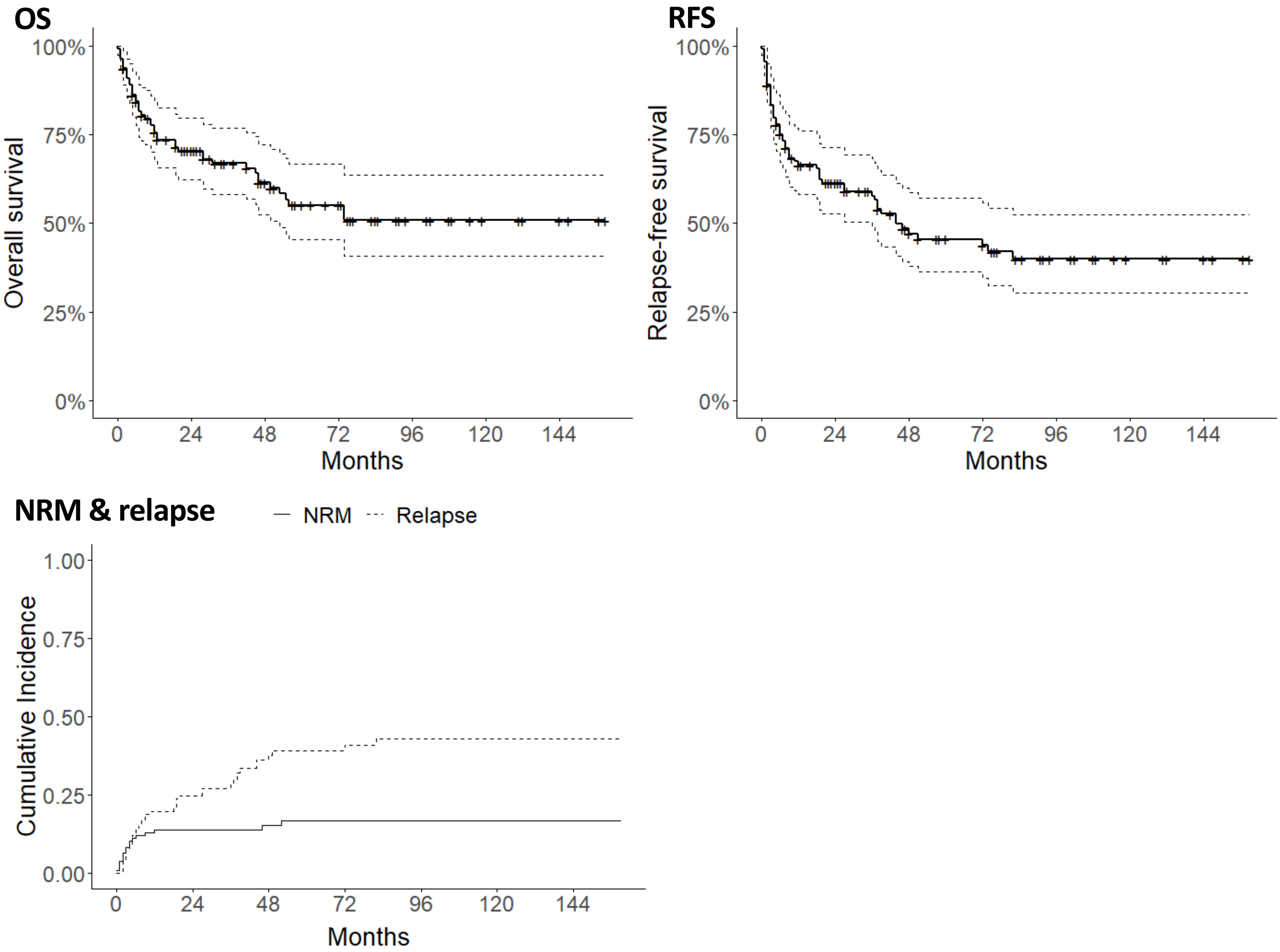

Figure S3: Long-term alloHCT outcomes.

Kaplan-Meier plots for long-term outcome variables of the entire cohort, median follow-up duration 63 months. Non-relapse mortality (NRM) and relapse incidence were analyzed as competing risks. 5-year overall survival (OS) 55% [95% CI 45.3-66.8%], relapse-free survival (RFS) 44% [95% CI 34.7-55.8%], NRM 17% [95% CI 10-25%], and relapse incidence 39% [95% CI 29-49%], n=109.

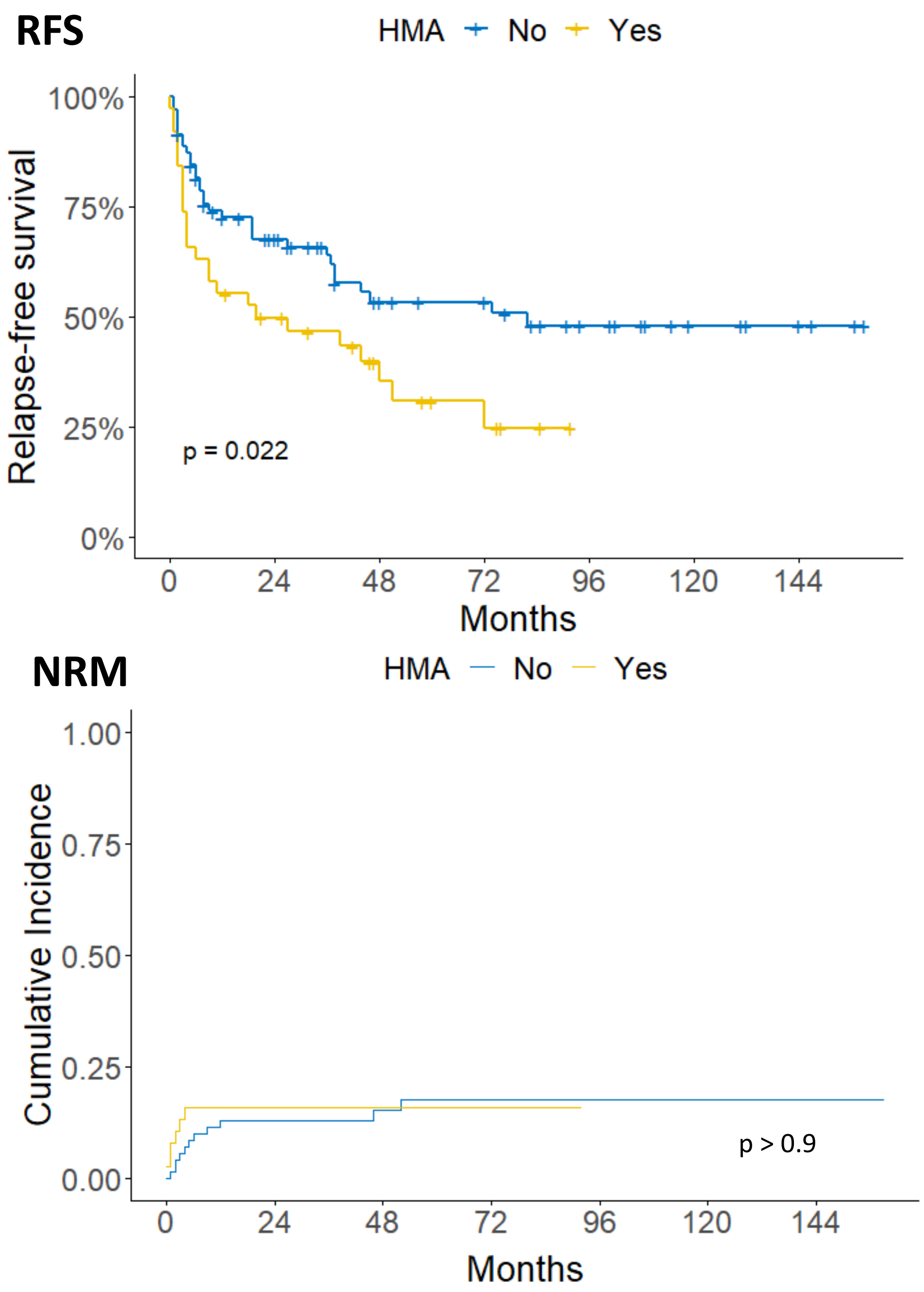

Figure S4: RFS and NRM after remission induction with HMA.
Comparison of time-dependent outcome variables according to remission induction with hypomethylating agents (HMA) using Kaplan-Meier estimator and log-rank test. Non-relapse mortality (NRM) and relapse incidence were analyzed as competing risks and tested for significant differences between groups by Gray‘s test. 5-year values for the HMA vs. non-HMA group: relapse-free survival (RFS) 30.9% vs. 53.4% (p=0. 022), NRM 16% vs. 18% (p>0.9), n=109.

Overall survival		
**Parameter**	**Strata**	**Multivariate HR (95% CI, p value)**
BM blasts	<5 %	-
	5 - 9%	1.62 (0.69-3.82, p=0.273)
	10 - 19%	1.13 (0.52-2.44, p=0.765)
Cytogenetic risk	Good	-
	Intermediate	2.46 (1.07-5.62, **p=0.033**)
	Poor	6.06 (2.49-14.74, **p<0.001**)
	Very poor	5.49 (2.30-13.09, **p<0.001**)
HMA	No	-
	Yes	2.06 (1.08-3.95, **p=0.029**)

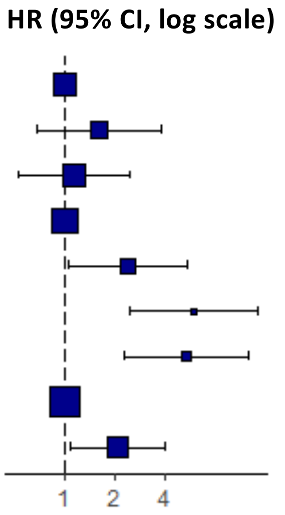

Relapse-free survival		
**Parameter**	**Strata**	**Multivariate HR (95% CI, p value)**
BM blasts	<5 %	-
	5 - 9%	1.29 (0.60-2.75, p=0.511)
	10 - 19%	1.15 (0.59-2.26, p=0.683)
Cytogenetic risk	Good	-
	Intermediate	2.12 (1.05-4.26, **p=0.036**)
	Poor	4.06 (1.87-8.78, **p<0.001**)
	Very poor	4.12 (1.93-8.79, **p<0.001**)
HMA	No	-
	Yes	1.95 (1.09-3.49, **p=0.025**)

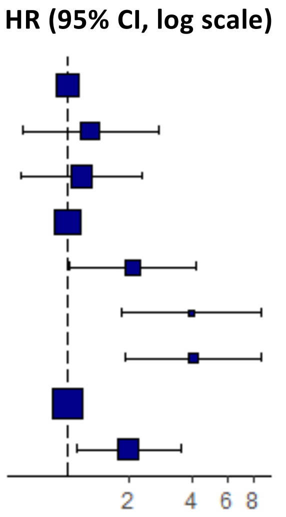

Relapse		
**Parameter**	**Strata**	**Multivariate HR (95% CI, p value)**
BM blasts	<5 %	-
	5 - 9%	1.26 (0.49-3.24, p=0.640)
	10 - 19%	1.77 (0.73-4.29, p=0.200)
Cytogenetic risk	Good	-
	Intermediate	2.68 (1.19-6.05, **p=0.018**)
	Poor	1.73 (0.61-4.92, p=0.310)
	Very poor	6.69 (2.87-15.61, **p<0.001**)
HMA	No	-
	Yes	2.09 (1.05-4.15, **p=0.035**)
Donor	Matched	-
	Mismatched	0.38 (0.13-1.11, p=0.077)

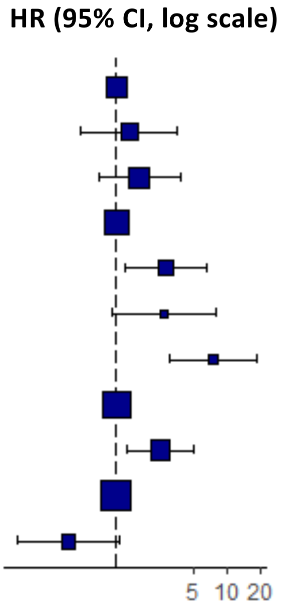

Figure S5: Multivariate regression analysis.
Visualization of hazard ratios (HR) and 95% confidence intervals (CI) for the predictive strength of multiple patient and treatment parameters towards overall survival (OS), relapse-free survival, and relapse incidence in the entire cohort. OS and RFS were analyzed by multivariate Cox Proportional Hazards regression analysis, relapse incidence was analyzed by a multivariate cause-specific hazards model. BM blasts, bone marrow blast percentage before initiation of treatment. HMA, hypomethylating agents. N=109.

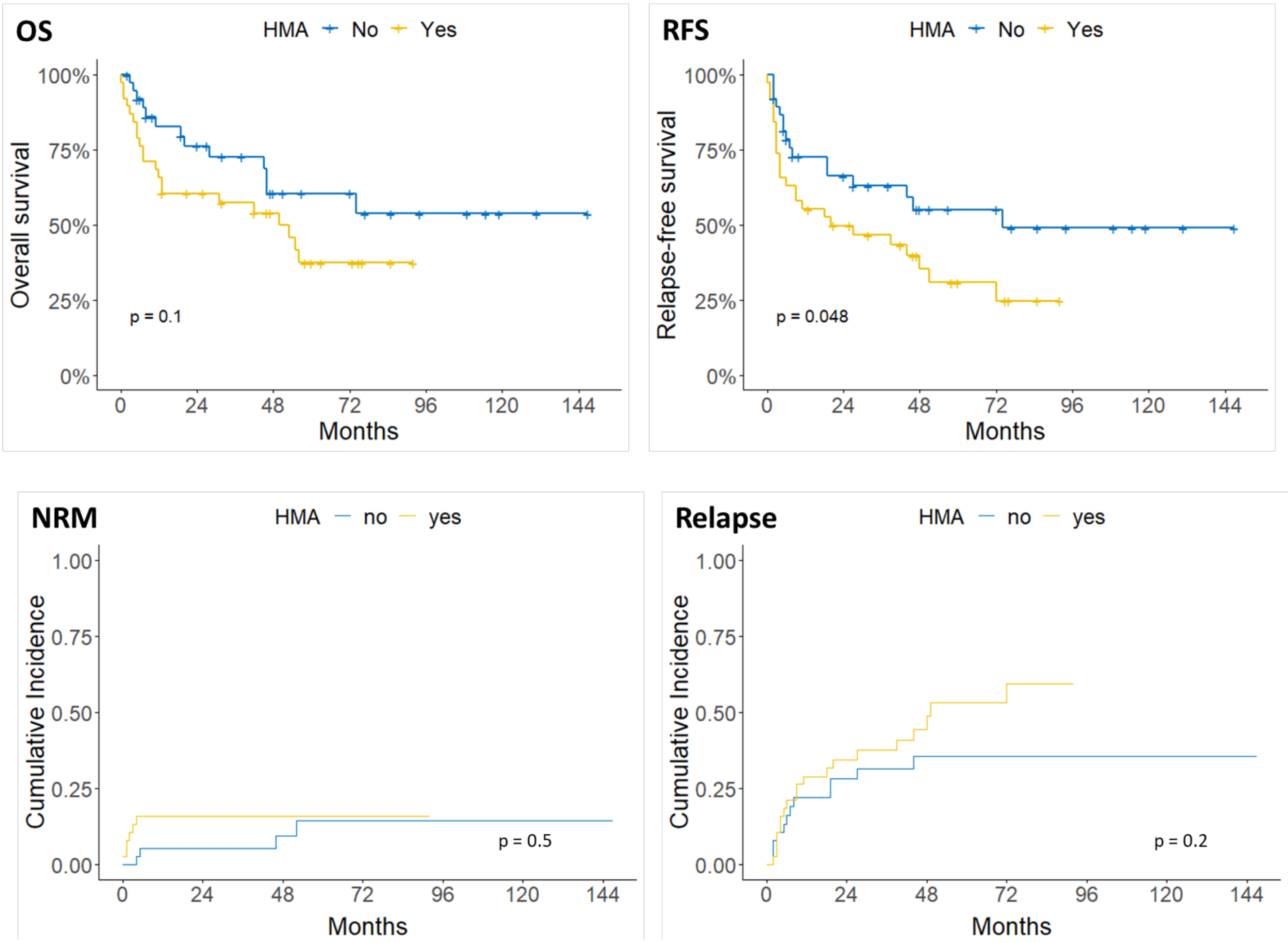

Figure S6: Propensity score matching.

Propensity score matching was performed using 1:1 nearest neighbor matching based on patient age, karnofsky index, and bone marrow (BM) blast count at diagnosis, resulting in 2 matched groups á 38 patients. Time-dependent outcome variables were then analyzed according to remission induction with hypomethylating agents (HMA) using Kaplan-Meier estimator and log-rank test. Non-relapse mortality (NRM) and relapse incidence were analyzed as competing risks and tested for significant differences between groups by Gray‘s test. 5-year values for the HMA vs. non-HMA group: Overall survival (OS) 37.4% vs. 60.5% (p=0.1), relapse-free survival (RFS) 30.9% vs. 55.1% (p=0.048), NRM 14% vs. 16% (p=0.5), and relapse incidence 53% vs. 36%, (p=0.2), n=76.

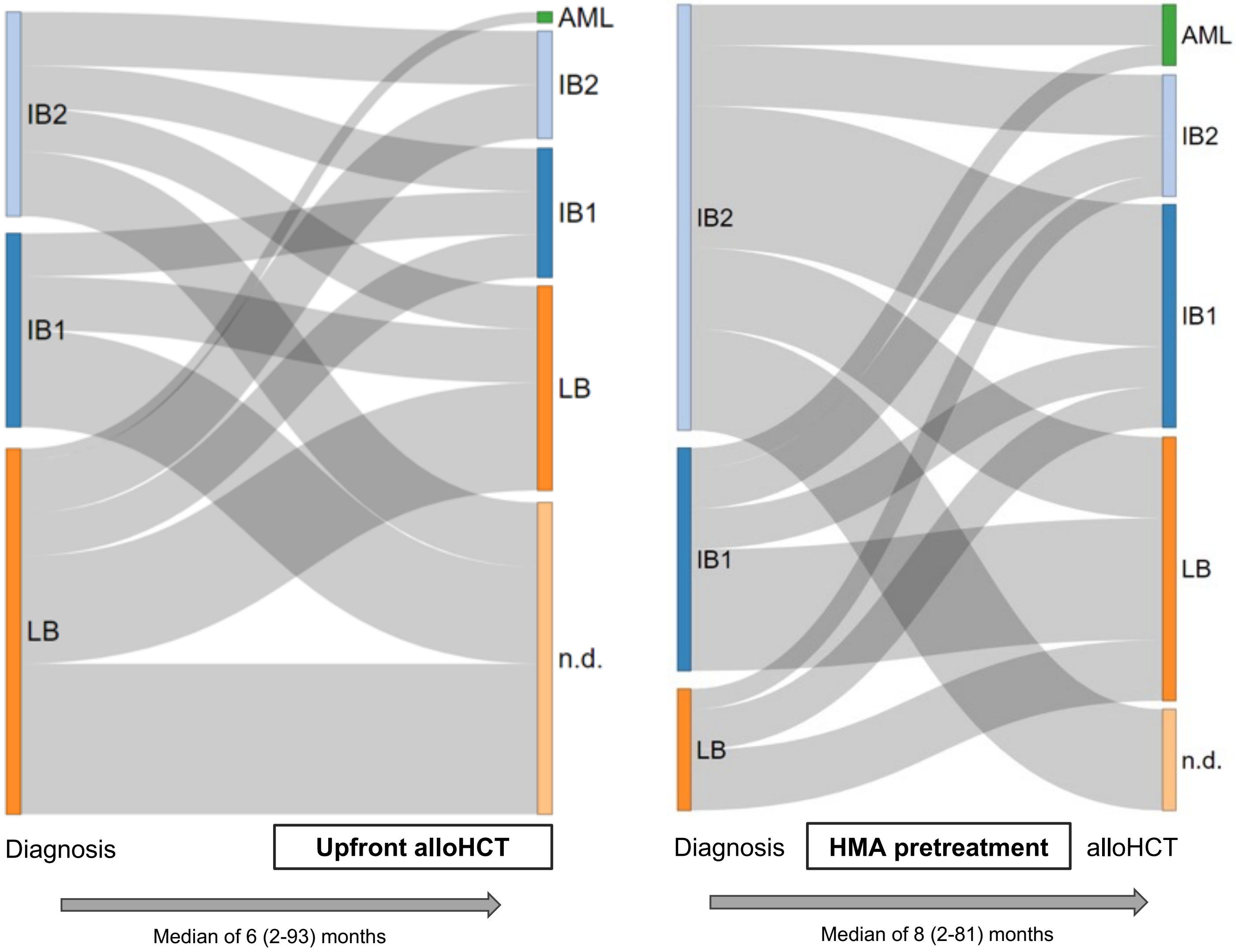

Figure S7: Changes in BM blast counts between diagnosis and alloHCT.
Sankey plot depicting the proportion of patients with bone marrow (BM) blast count diagnostic categories for myelodysplastic syndrome (MDS) low blasts (LB), increased blasts (IB) 1 and 2, and acute myeloid leukemia (AML), respectively indicating blast counts of <5%, 5-9%, 10-19%, or ≥ 20% both at diagnosis and at allogeneic hematopoietic cell transplantation (alloHCT) depending on pre-treatment strategy with hypomethylating agents (HMA) versus upfront alloHCT. N=109, BM blast count evaluation at alloHCT was not done (n.d.) in 34 of 109 (31.2%) patients.
